# Zinc-finger protein 418 overexpression protects against cardiac hypertrophy and fibrosis

**DOI:** 10.1371/journal.pone.0186635

**Published:** 2017-10-24

**Authors:** Liming Pan, Mengting Sheng, Zirui Huang, Zhilin Zhu, Chunli Xu, Lin Teng, Ling He, Chen Gu, Cai Yi, Junming Li

**Affiliations:** 1 Department of Cardiology, the People’s Hospital of Three Gorges University/the First People’s Hospital of Yichang, Yichang, China; 2 Department of Intensive Care Unit(ICU), the People’s Hospital of Three Gorges University/the First People’s Hospital of Yichang, Yichang, China; 3 Department of Inspection office, the People’s Hospital of Three Gorges University/the First People’s Hospital of Yichang, Yichang, China; 4 Department of Cardiology, the First College of Clinical Medical Sciences of Three Gorges University/ Central People's Hospital of Yichang, Yichang, China; 5 Department of Geriatrics, the People’s Hospital of Three Gorges University/the First People’s Hospital of Yichang, Yichang, China; 6 Department of B ultrasound room, the People’s Hospital of Three Gorges University/the First People’s Hospital of Yichang, Yichang, China; 7 Institute of Cardiovascular Diseases, China Three Gorges University, Yichang, China; Max Delbruck Centrum fur Molekulare Medizin Berlin Buch, GERMANY

## Abstract

**Background:**

This study aimed to investigated the effect and mechanism of zinc-finger protein 418 (ZNF418) on cardiac hypertrophy caused by aortic banding (AB), phenylephrine (PE) or angiotensin II (Ang II) *in vivo* and *in vitro*.

**Methods:**

The expression of ZNF418 in hearts of patients with dilated cardiomyopathy (DCM) or hypertrophic cardiomyopathy (HCM) and AB-induced cardiac hypertrophy mice, as well as in Ang II- or PE-induced hypertrophic primary cardiomyocytes was detected by western blotting. Then, the expression of ZNF418 was up-regulated or down-regulated in AB-induced cardiac hypertrophy mice and Ang II -induced hypertrophic primary cardiomyocytes. The hypertrophic responses and fibrosis were evaluated by echocardiography and histological analysis. The mRNA levels of hypertrophy markers and fibrotic markers were detected by RT-qPCR. Furthermore, the phosphorylation and total levels of c-Jun were measured by western blotting.

**Results:**

ZNF418 was markedly down-regulated in hearts of cardiac hypertrophy and hypertrophic primary cardiomyocytes. Down-regulated ZNF418 exacerbated the myocyte size and fibrosis, moreover increased the mRNA levels of ANP, BNP, β-MHC, MCIP1.4, collagen 1a, collagen III, MMP-2 and fibronection in hearts of AB-treated ZNF418 knockout mice or Ang II-treated cardiomyocytes with AdshZNF418. Conversely, these hypertrophic responses were reduced in the ZNF418 transgenic (TG) mice treated by AB and the AdZNF418-transfected primary cardiomyocytes treated by Ang II. Additionally, the deficiency of ZNF418 enhanced the phosphorylation level of c-jun, and overexpression of ZNF418 suppressed the phosphorylation level of c-jun *in vivo* and *in vitro*.

**Conclusion:**

ZNF418 maybe attenuate hypertrophic responses by inhibiting the activity of c-jun/AP-1.

## Introduction

Cardiac hypertrophy is a common pathological change in patients with progressive cardiac function failure, which can be caused by some intrinsic or extrinsic factors, including familial hypertrophic cardiomyopathy (HCM) or arterial hypertension and dilated cardiomyopathy (DCM) [1,2]. Although cardiac hypertrophy is considered as an adaptive response to hemodynamic and neurohormonal stress, prolonged hypertrophy can result in functional decompensation, arrhythmias and cardiac fibrosis, even progress to heart failure or sudden death [2–4]. Therefore, it is urgent to in-depth understand the underling mechanism of cardiac hypertrophy and search for effective therapeutic targets for cardiac hypertrophy.

A variety of pathological stimuli such as pressure overload, angiotensin II (Ang II) and phenylephrine (PE) have reported to induce cardiac hypertrophy. Cardiac hypertrophy not only can show increased cell size and collagen synthesis, but also induce enhanced expressions of the relevant fetal genes, including atrial natriuretic peptide (ANP), brain natriuretic peptide (BNP), β-myosin heavy chain (β-MHC) and modulatory calcineurin-interacting protein 1.4 (MCIP1.4) [5–7]. Previous studies have demonstrated that a number of signal transduction pathways are implicated in the development of cardiac hypertrophy, including mitogen-activated protein kinase (MAPK) pathway, phosphoinositide 3-kinase (PI3K)/Akt pathway, protein kinase C (PKC) pathway [8–10]. Notably, these intracellular signaling cascades can regulate the expression levels of ANP, BNP, and β-MHC through activating various transcriptional regulatory proteins such as activator protein-1 (AP-1), thereby exacerbating cardiac hypertrophy [7,11].

The krüppel-associated box/Cys2His2 (KRAB/C2H2) zinc finger proteins are considered as the largest group of transcription factors known in vertebrates, which have been proved to be involved in tumor suppression, neoplastic transformation, cell differentiation, proliferation and apoptosis [12–14]. ZNF418, a novel human KRAB/C2H2 zinc finger gene form a human embryo heart cDNA library is identified. The gene spans 13.5 kb on chromosome 19q13.43 encompassing six exons, and transcribes a 3.7-kb mRNA that encodes a protein with 676 amino acid residues. The ZNF418 protein expressed in various adult tissues, including the placenta, pancreas, kidney, liver, brain, muscle, lung, and heart [15]. Interestingly, up-regulation of ZNF418 is reported to inhibit the transcriptional expression of AP-1 [15]. An increasing number of researches have supported that down-regulated AP-1 protects ventricular cardiomyocytes against the induction of hypertrophy and apoptosis [16–19], indicating that ZNF418 may participate in the occurrence and development of cardiac hypertrophy. However, the effect of ZNF418 on cardiac hypertrophy has not previously been investigated.

In the present study, we observed the expression of ZNF418 in hearts of DCM or HCM patients and aortic banding (AB)-induced cardiac hypertrophy mice *in vivo*, as well as in Ang II- or PE-induced hypertrophic primary cardiomyocytes *in vitro*. Then, the expression of ZNF418 was up-regulated or down-regulated in AB-induced cardiac hypertrophy mice and Ang II -induced hypertrophic primary cardiomyocytes, aimed to explore the effect of ZNF418 on cardiac hypertrophy *in vivo* and *in vitro*. Furthermore, we investigate whether AP-1 subunit c-Jun was involved in the development of cardiac hypertrophy regulated by ZNF418.

## Materials and methods

### Ethics statement

This study was approved by the Human Research Ethics Committee of China Three Gorges University People’s Hospital. All the human heart samples were collected after written informed consent by patients. Approval from the Laboratory Animal Management Committee of Three Gorges University was obtained prior to all animal experiments.

### Human heart samples

Our research team and Cardiovascular Research Institute of Wuhan University share DCM or HCM hearts provided by Renmin Hospital of Wuhan University [20]. And, none of the transplant donors were from a vulnerable population and all donors or next of kin provided written informed consent that was freely given. All human heart samples used by our study were separated from the left ventricles of patients with DCM or HCM who underwent heart transplantation. The normal heart samples were collected from the donors who died in noncardiac reasons with normal cardiac function.

### Isolation of primary cardiomyocytes and cell treatment

Primary cardiomyocytes were isolated from healthy Sprague-Dawley rats (1- to 2-day-old, provided by the Animal Laboratory Center of Three Gorges University, Yichang, China). In brief, the ventricular myocardium was cut into small pieces and then incubated in phosphate buffered saline (PBS) containing 0.04% collagenase type II and 0.03% trypsin for 8 min at 37°C. The fibroblasts were removed and the primary cardiomyocytes were collected using a differential attachment technique. The collected primary cardiomyocytes were resuspended with Dulbecco's modified eagle medium (DMEM)/F12 (Gibco, GrandIsland, NY, USA) containing 20% fetal bovine serum, 0.1 mM BrdU, 100 units/ml penicillin and 100 mg/ml streptomycin, and then placed onto collagen-coated 6-well plates at a density of 1×10^6^ cells/well. After 48 h of incubation, the cell culture medium was then changed to serum-free DMEM/F12 for 12 h and the primary cardiomyocytes were treated with phenylephrine (PE, 100 μM) or angiotensin II (Ang II, 1 μM) for 48 h to induce cardiomyocytes hypertrophy. The ZNF418 gene overexpression recombinant adenovirus was constructed and named as AdZNF418. The full-length rat ZNF418 cDNA under control of the cytomegalovirus promoter was cloned into a replication-defective adenoviral vector. A similar adenoviral vector expressing green fluorescent protein (GFP) was used as a control (named as AdGFP). The ZNF418 gene silence recombinant adenovirus was constructed and named as named as AdshZNF418. The ZNF418 shRNA (target sequence: AGAGCCAAGAATTGGTTAAAG) was cloned into pRNTR-U6 adenoviral vector. A blank adenoviral vector (named as AdshRNA) were was used as a control. Sequence analysis confirmed that AdZNF418 and AdshRNA sequences were correct. Then, the successfully constructed adenoviral vector and packaging plasmid (mix) were co-transfected into the 293T cells using Lipofectamine 2000 (Invitrogen Life Technologies, Carlsbad, CA, USA). Recombinant adenoviruses were generated by packaging and purified by velocity density gradient centrifugation in caesium chloride solutions. The primary cardiomyocytes were infected with recombinant adenoviruses at a multiplicity of infection of 100 for 24 h, respectively.

### Animal models

Cardiac-specific ZNF418 knockout (KO) mice, Cardiac-specific ZNF418 transgenic (TG) mice and wild-type (WT) littermates (male, aged 8–10 weeks, body weight of 23–26 g) were purchased from the Animal Care and Use Committee of Renmin Hospital of Wuhan University. All mice were housed in temperature-controlled cages under a 12-hour light–dark cycle and given free access to water and normal rodent chow. Cardiac hypertrophy mouse models in ZNF418 KO mice (n = 21), ZNF418 TG mice (n = 13) and WT mice (n = 26) were established by AB operation. Briefly, mice were anesthetized with sodium pentobarbital (80 mg/kg) by intraperitoneal injection, and then given ventilator-assisted breathing (rate of 70 beats/min, VE of 1.0~1.5 ml/min). The left chest of each mouse was opened by blunt dissection at the second-three intercostal space to recognize the thoracic aorta. Next, the aorta descendens was tied using 6–0 silk sutures against a 27-gauge needle, followed by removed the needle and closed the thoracic cavity. The sham operation in ZNF418 KO mice (n = 12), ZNF418 TG mice (n = 13) and WT mice (n = 25) were carried out with a similar procedure, but without constricting. After AB treatment for 4 weeks, the hearts and lungs of the mice were harvested and weighed, and the tibial lengths were measured. The ratios of the heart weight/body weight (HW/BW, mg/g), lung weight/bodyweight (LW/BW, mg/g) and heart weight/tibial length (HW/TL, mg/cm) were then calculated. The hearts of mice were kept at −80°C for protein and RNA analysis or fixed in 10% formalin for 24 h. All experiments were performed in accordance with the criteria of the Guide for the Care and Use of Laboratory Animals published by the National Institutes of Health. If we find experiment mouse have dyspnea symptom, with the diagnosis of Transthoracic echocardiography suggested heart function decreased significantly. Then they will be fed isolated for reducing the probability of infection. If the physical condition of the mouse becomes worse and cannot reach the experiment endpoint. We will perform euthanasia on these mice. As a result, there were 7 mice were euthanized which suffered heart failure after AB surgery.

### Generation of ZNF418 knockout (ZNF418-KO) mice

We predicted the guide sequences that target the exon 2 of ZNF418 gene in the mouse genome using the online CRISPR design tool (http://crispr.mit.edu; S1A Fig). The paired synthesized oligonucleotides (oligo1: TAGGAAAAGGGTATGGTCTGTATAC and oligo 2: AAACGTATACAGACCATACCCTTTT) were annealed and cloned into the pUC57-sgRNA expression vector (Addgene 51132). The result plasmid was used for *in vitro* transcription using a MEGA shortscript Kit (Ambion, AM1354). A Cas9 expression plasmid (Addgene 44758) was linearized and served as the template for *in vitro* transcription using the T7 Ultra Kit (Ambion, AM1345). Both the transcribed Cas9 mRNA and sgRNA were injected into fertilized mice eggs using a FemtoJet 5247 microinjection system under standard conditions. Genomic DNA obtained from a mouse tail biopsy was extracted and used for founder identification. A portion of the ZNF418 gene spanning the target site was amplified by PCR using the primers ZNF418-F (5’- AAGCTTTGTGCAACATCCCG -3’) and ZNF418-R (5’- CCAACTGAGCTATCTTATCAATCAC -3’). The PCR products were sequenced directly to allow the identification of editing events (S1B Fig). Also, T-A colonies cloned from PCR products from the founders were sequenced (S1C Fig).Primers ZNF418-137-F (5’-GGTCCAGGTCCATGAAAACT-3’) and ZNF418-137-R (5’- TGGCCACACTGGTGAACTTA-3’) were used to screening F1 and F2 offspring. The products were analyzed by 3.0% agarose gel electrophoresis in TAE buffer. The wildtype allele yielded an amplicon of 137 bp, while the mutant allele yielded an amplicon of 129 bp (S1D Fig).

### Generation of ZNF418 transgenic (ZNF418-TG) mice

We initially generated TG mice carrying a construct of the CAG gene promoter-loxP-CAT gene-loxP-mZNF418 region (designated CAG-CAT-mZNF418 TG mice). Founder TG mice were identified by tail DNA amplification and then bred with C57BL/6J mice. The PCR primers used for TG detection were CAG-forward (5’-CCCCCTGAACCTGAAACATA-3’) and ZNF418-reverse (5’- CCTTCTTGGGAGACACACCT-3’), which yielded a 580-bp product. When the CAG-CAT-mZNF418-TG mice were crossed with α-MHC-MerCreMer-TG mice (Myh6-cre/Esr1, Jackson Laboratory, 005650), the tamoxifen-inducible Cre-mediated recombination was expected to result in the deletion of CAT gene, leading to the expression of mZNF418 specifically in the myocardium of the offspring. The CAG-CAT-mZNF418/α-MHC-MerCreMer mice at 6 weeks of age were injected intraperitoneally with tamoxifen (Sigma-Aldrich, T5648, 25 mg/kg per day) for 5 consecutive days to generate cardiac-specific ZNF418-overexpressed mice.

### Echocardiography and hemodynamics

Transthoracic echocardiography was performed using a Mylab30CV machine (Biosound ESAOTE Inc., Indianapolis, IN) with a 10-MHz linear array ultrasound transducer. Then left ventricle end-diastolic dimension (LVEDD), left ventricle end-systolic diameter (LVESD), ejection fraction (EF) and fraction shortening (FS) of left ventricle were measured. The mean values were obtained from at least three different cardiac cycles.

### Histological analysis and immunohistochemistry

The fixed hearts were embedded into paraffin blocks, and then sliced transversely into 5 μm-thick sections. Next, sections were dipped into gradient ethanol, and then stained with picrosirius red (PSR, Shanghai Jianglai Biotechnology Co., Ltd, China) to detect collagen fibers or with hematoxylin-eosin (HE, Shanghai Jianglai Biotechnology Co., Ltd) to observe the cell cross-sectional area. In addition, to intuitively observe the cross-sectional areas of the myocytes, the sections were stained with fluorescein isothiocyanate (FITC)-conjugated wheat germ agglutinin (WGA, Invitrogen, Carlsbad, CA, USA). All the sections were imaged by BX51 light microscopy (Olympus, Tokyo, Japan) or TE2000 inversed fluorescence microscope (Nikon, Tokyo, Japan). The size of a single myocyte was measured using a quantitative digital image analysis system (Image-Pro Plus 6.0, Media Cybernetics Inc., Silver Spring, MD, USA).

### Cell immunofluorescence analysis

After infection with ZNF418-overexpressing adenovirus (AdZNF418) or ZNF418-interfering adenovirus (AdshZNF418) for 24 h, the primary cardiomyocytes were treated with Ang II (1 μM) for 48 h. Then the cardiomyocytes were fixed with 4% paraformaldehyde for 15 min, followed by permeabilized with 0.2% Triton X-100 in PBS for 40 min. Subsequently, the cells were blocked with 1% goat serum, immunostained with anti-α-actinin monoclonal antibody (Sigma, St. Louis, MO, USA), and incubated with FITC labeled IgG second antibody (Wuhan BosterBio Co., China). The cell nucleus were stained with DAPI (Wuhan BosterBio Co.) for 5 min. Finally, the cells were imaged by TE2000 inversed fluorescence microscope and analyzed by Image-Pro Plus 6.0 analysis software.

### Real-time quantitative PCR (RT-qPCR)

Total RNA from frozen cardiac tissue or the primary cardiomyocytes was extracted using TRIZol reagent (Invitrogen), and then complementary DNA was synthesized using the Transcriptor First Strand cDNA Synthesis Kit (Roche, Basel, Switzerland). The indicated gene transcript levels were measured by PCR amplification using the LightCycler 480 SYBR Green I Master kit (Roche). The specific primers for glyceraldehyde-3-phosphate dehydrogenase (GAPDH), ANP, BNP, β-MHC, MCIP1.4, Collagen 1a, Collagen III, matrix metalloproteinase-2 (MMP-2) and Fibronection are listed in Table 1. The relative mRNA levels of these genes were calculated by comparative threshold (*C*t) cycle method (2^–ΔΔ^*C*t) and normalized with GAPDH mRNA.

**Table 1:** Primer sequences for specific genes.

Gene	Primer sequence
ANP	F: 5'-ACCTGCTAGACCACCTGGAG-3'
	R: 5'-CCTTGGCTGTTATCTTCGGTACCGG-3'
BNP	F: 5'-GAGGTCACTCCTATCCTCTGG-3'
	R: 5'-GCCATTTCCTCCGACTTTTCTC-3'
β-MHC	F:5'-CCGAGTCCCAGGTCAACAA-3'
	R: 5'-CTTCACGGGCACCCTTGGA-3'
MCIP1.4	F: 5'-TCCAGCTTGGGCTTGACTGAG-3'
	R: 5'-ACTGGAAGGTGGTGTCCTTGT-3'
Collagen1α	F: 5'-AGGCTTCAGTGGTTTGGATG-3'
	R: 5'-CACCAACAGCACCATCGTTA-3'
CollagenIII	F: 5'-CCCAACCCAGAGATCCCATT-3'
	R: 5'-GAAGCACAGGAGCAGGTGTAGA-3'
Fibronecin	F: 5'-CCGGTGGCTGTCAGTCAGA-3'
	R: 5'-CCGTTCCCACTGCTGATTTATC-3'
MMP-2	F: 5'-TTTGCTCGGGCCTTAAAAGTAT-3'
	R: 5'-CCATCAAACGGGTATCCATCTC-3'
GAPDH	F: 5'-ACTCCACTCACGGCAAATTC-3'
	R: 5'-TCTCCATGGTGGTGAAGACA-3'

ANP, atrial natriuretic peptide; BNP, brain natriuretic peptide; β-MHC, β-myosin heavy chain; MCIP1.4, modulatory calcineurin-interacting protein1.4;MMP-2, matrix metalloproteinase-2; GAPDH, glyceraldehyde-3-phosphate dehydrogenase.

### Western blotting

Total protein was extracted by homogenizing the heart tissue or the primary cardiomyocytes in lysis buffer (Beyotime Institute of Biotechnology, China) for 1.5 h on ice. The protein concentration was measured using a BCA Protein Assay kit (Beyotime Institute of Biotechnology). Subsequently, 50 μg of protein sample was resolved through SDS–PAGE gel (Invitrogen), and the resolved proteins were blotted onto a PVDF membrane (Millipore, Bedford, MA, USA). The membranes were blocked with 10% non-fat milk for 1h at room temperature, followed by probed with anti-ANP, β-MHC, ZNF418, GAPDH, c-jun and phosphorylated-c-jun (p-c-jun) primary antibodies (Santa Cruz, Santa Cruz, CA,USA) overnight at 4°C and incubated a IRDye 800CW-conjugated secondary antibody (LI-COR Biosciences, Lincoln, NE, USA) at 1:10,000 dilution for 1h at room temperature. Ultimately, the protein expressions were detected by Enhanced chemiluminescence (Millipore) and visualized using an Odyssey Imaging System (LI-COR Biosciences).

### Statistical analysis

IBM SSPS statistics 20.0 software (SPSS Inc., Chicago, IL, USA) was used to analyze data. Continuous variables were represented as the mean ± S.D. And all data analysis were performed using Student’s two-tailed *t*-test or ANOVA followed by LSD or Tamhane’s test. Values of *P* ˂ 0.05 were considered to be statistically significant.

## Results

### ZNF418 is down-regulated in hearts of patients with DCM or HCM as well as cardiac hypertrophy mice

**Fig 1:**
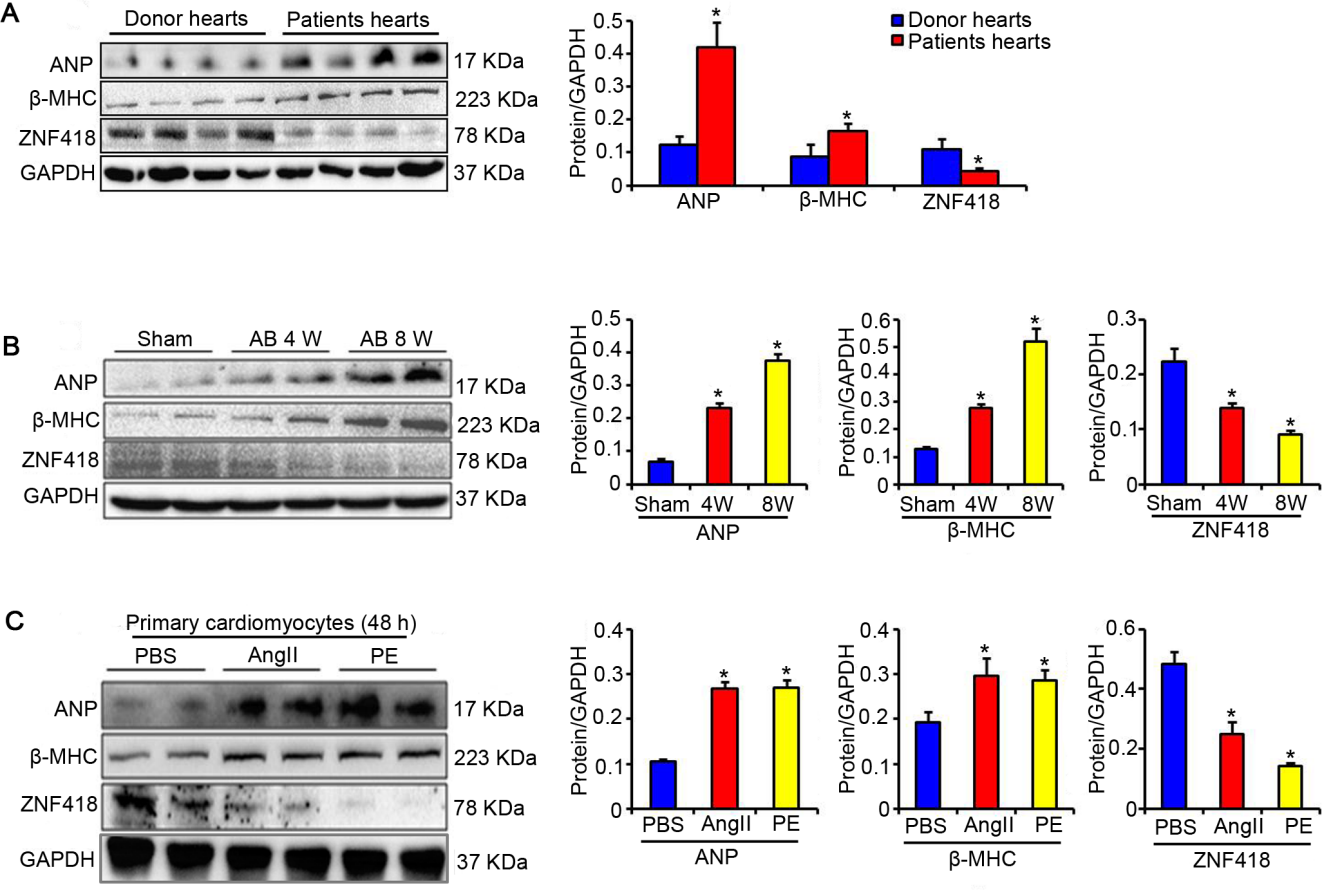
ZNF418 expression is decreased in hearts of patients withdilated cardiomyopathy (DCM) or hypertrophic cardiomyopathy (HCM) as well as cardiac hypertrophy mice. **(A)** Expression levels of atrial natriuretic peptide (ANP), β-myosin heavy chain (β-MHC) and Zinc-finger protein 418 (ZNF418) in donor hearts and patients hearts by western blotting analysis (n = 4 samples per group, *P < 0.05 vs. donor hearts); **(B)** The protein levels of ANP, β-MHC and ZNF418 in hearts of wild-type mice after sham or aortic binding (AB) surgery by western blotting analysis (n = 4 mice per group, *P < 0.05 vs. sham hearts); **(C)** Expression levels of ANP, β-MHC and ZNF418 in primary cardiomyocytes treated with angiotensin II (Ang II, 1 μM) or phenylephrine (PE, 100 μM), or phosphate buffered solution (PBS) for 48 h by western blotting analysis (n = 3 samples per group, *P < 0.05 vs. PBS).

To explore the effect of ZNF418 on pathological cardiac hypertrophy, we first detected the expression of ZNF418 in hearts of patients with DCM or HCM as well as hearts of cardiac hypertrophy mice. Western blotting results showed that compared with donor hearts, ZNF418 protein expression was significantly decreased in hearts of patients with DCM or HCM (*P* < 0.05, Fig 1A). In addition, the protein levels of hypertrophic markers, including ANP and β-MHC, were remarkably up-regulated in hearts of patients with DCM or HCM compared with donor hearts (*P* < 0.05, Fig 1A). Consistently, after AB treatment for 4 weeks, the expression of ZNF418 was also lower, but ANP and β-MHC levels were higher in hearts of cardiac hypertrophy mice than hearts of sham mice (*P* < 0.05, Fig 1B), and more significant changes were observed at 8week (*P* < 0.05, Fig 1B). Furthermore, after treatment with either Ang II or PE for 48 h, the expression level of ZNF418 was reduced by 47% (Ang II) and 71% (PE) compared with primary cardiomyocytes treated with PBS (*P* < 0.05, Fig 1C). Primary cardiomyocytes treated with Ang II or PE showed higher levels of ANP and β-MHC than cells treated with PBS (*P* < 0.05, Fig 1C), indicated that cardiomyocytes hypertrophy model was successfully induced by Ang II or PE.

### ZNF418 suppresses Ang II-induced cardiomyocyte hypertrophy *in vitro*

**Fig 2:**
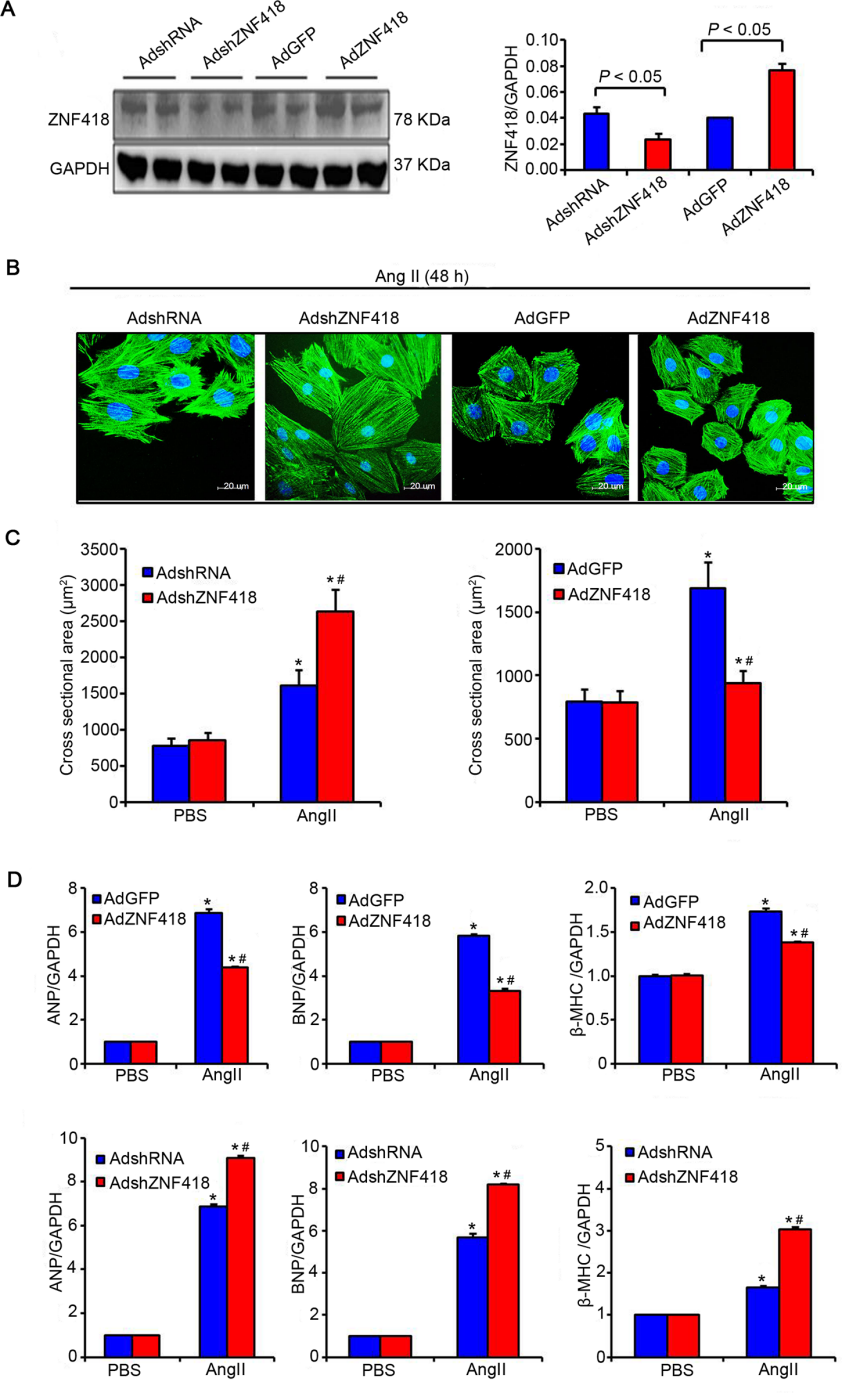
ZNF418 overexpression suppresses angiotensin II (Ang II)–induced cardiomyocyte hypertrophy. **(A)** Primary cardiomyocytes infected with AdZNF418, AdshZNF418, or their respective controls (AdGFP and AdshRNA) were analyzed by western blotting (n = 3 independent experiments, *P < 0.05 vs. AdGFP or shRNA); **(B)** Representative images of cardiomyocytes that have been infected with AdshZNF418 or AdZNF418 after treatment with Ang II (1 μM) for 48 h by the immunofluorescence analysis of α-actinin. Blue: nuclear; Green: α-actinin; Scale bar, 20 μm; **(C)** Cell surface area of cardiomyocytes that have been infected with AdshZNF418 (left) or AdZNF418 (right), as well as treated with Ang II for 48 h (n = 4 independent experiments, *P < 0.05 vs. AdGFP, AdZNF418, AdshRNA or AdshZNF418/PBS, ^#^*P* < 0.05 vs. AdGFP or AdshRNA/Ang II); **(D)** The results of Real-time quantitative PCR showed the hypertrophy markers atrial natriuretic peptide (ANP), brain natriuretic peptide (BNP) and β-myosin heavy chain (β-MHC) mRNA levels in ZNF418 overexpressed (above) and knockdown (below) cardiomyocytes after PBS or Ang II treatment (n = 4 independent experiments, *P < 0.05 vs. AdGFP, AdZNF418, AdshRNA or AdshZNF418/PBS, ^#^*P* < 0.05 vs. AdGFP or AdshRNA/Ang II).

To explore the causal relationship between ZNF418 and cardiomyocyte hypertrophy, we examined the consequences of up-regulating or down-regulating ZNF418 level in primary cardiomyocytes. Western blotting results showed that the expression of ZNF418 was significantly up-regulated in cells with AdZNF418 compared with cells with AdGFP, conversely, markedly down-regulated in cells with AdshZNF418 compared with cells with AdshRNA (all *P* < 0.05, Fig 2A). After stimulated with Ang II or PBS control for 48h, the size of cardiomyocyte was measured by the immunofluorescence analysis of α-actinin (Fig 2B). The results showed that compared with Ang II-induced cells with AdGFP, the cell surface area of cardiomyocytes was obviously reduced in Ang II-induced cells with AdZNF418 (*P* < 0.05, Fig 2C), indicated that up-regulated ZNF418 suppressed Ang II-induced cardiomyocyte hypertrophy; in contrast, AdshZNF418 treatment enhanced Ang II–treatmented increase in cell size compared with AdshRNA treatment (*P* < 0.05, Fig 2C). Furthermore, Ang II-induced cells with AdZNF418 showed significantly lower mRNA levels of ANP, BNP and β-MHC than Ang II-induced cells with AdGFP, while AdshZNF418 treatment elevated the mRNA levels of ANP, BNP and β-MHC compared with AdshRNA treatment (all *P* < 0.05, Fig 2D).

### Deficiency in ZNF418 exacerbates AB-induced cardiac hypertrophy *in vivo*

**Fig 3:**
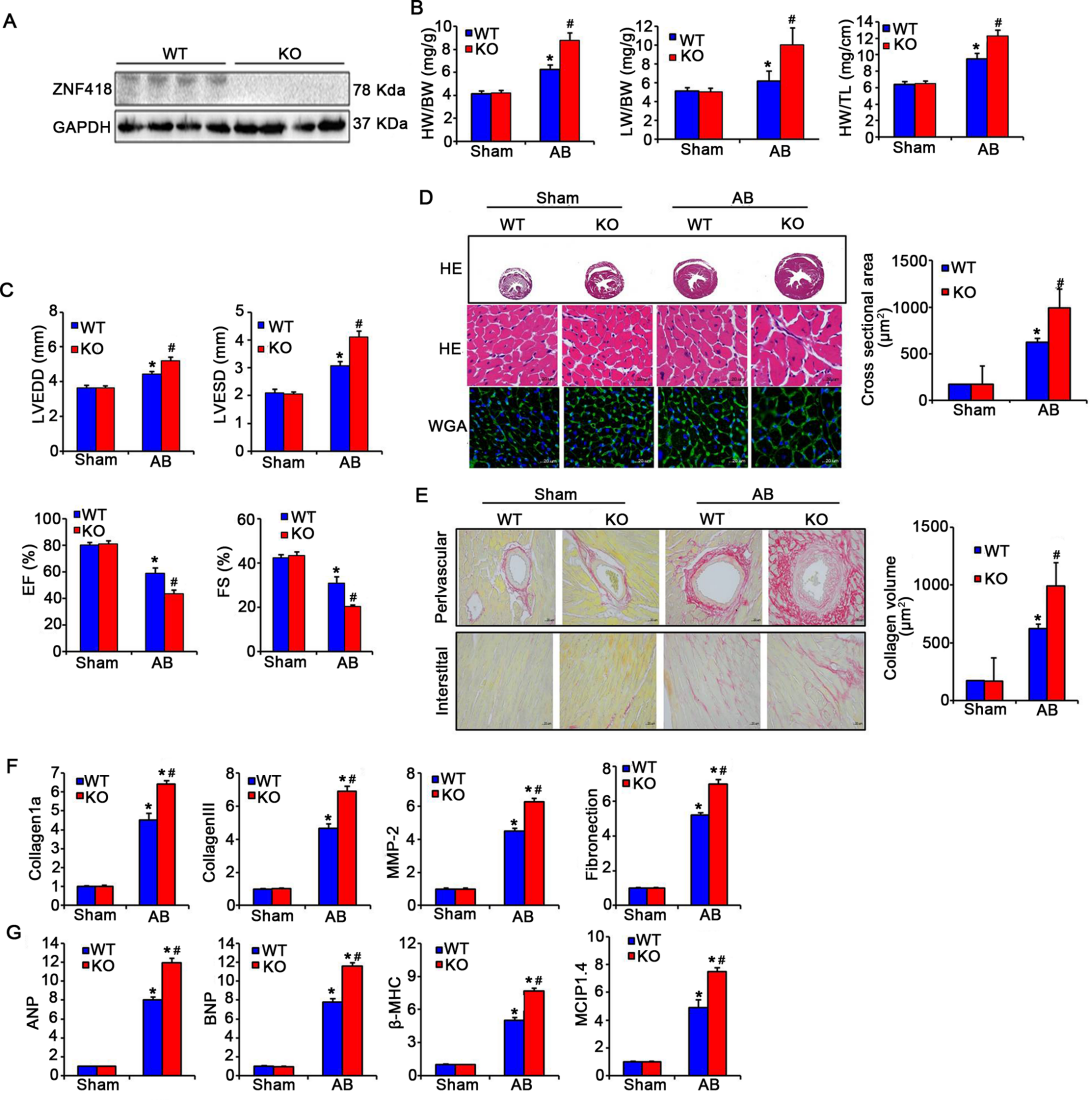
Loss of ZNF418 aggravates aortic banding (AB)-induced hypertrophy. **(A)** The expression of ZNF418 in the hearts form wild-type (WT) and ZNF418 knockout (KO) mice by western blotting; **(B)** Statistical results for the percentage of heart weight/body weight (HW/BW), lung weight (LW)/BW and HW/tibial length (TL) (n = 13 for each group, *P < 0.05 vs. WT/sham; ^#^*P* < 0.05 vs. WT/AB); **(C)** Statistical results for the parameters of the echocardiographic results, including left ventricle end-diastolic dimension (LVEDD), left ventricle end-systolic diameter (LVESD), ejection fraction (EF) and fraction shortening (FS) in WT and KO mice (n = 6–7 mice per experimental group); **(D)** Histological analyses of the cross sectional area of hearts form WT and ZNF418 KO mice after sham treatment or AB surgery for 4 weeks were stained with HE and WGA (n = 7 mice per group; scale bar, 100 μm for top HE staining, scale bar, 20 μm for middle HE staining and lower WGA staining); **(E)** Picrosirius red staining on histological sections from the left ventricles of WT and KO mice hearts after sham or AB surgery for 4 weeks (n = 7–8 mice per experimental group; Scale bars, 20 μm). The fibrotic areas of individual sections were quantified using Image-Pro Plus 6.0 software; **(F)** The mRNA expression levels of fibrotic markers-collagen 1a, collagen III, matrix metalloproteinase-2 (MMP-2), Fibronectin in the hearts of WT and KO mice were detected with real-time quantitative PCR after sham and AB surgery for 4 weeks (n = 5,*P < 0.05 vs. WT/sham or KO/sham, ^#^*P* < 0.05 vs. WT/AB); **(G)** The results of real-time quantitative PCR showed the mRNA levels of hypertrophy markers, including atrial natriuretic peptide (ANP), brain natriuretic peptide (BNP), β-myosin heavy chain (β-MHC) and modulatory calcineurin-interacting protein1.4 (MCIP1.4), in ZNF418KO and WT mice hearts after AB or sham treatment for 4 weeks (n = 5–6 independent experiments, *P < 0.05 vs. WT/sham or KO/sham, ^#^*P* < 0.05 vs. WT/AB).

To further to explore the effect of ZNF418 on cardiac hypertrophy *in vivo*, ZNF418 KO mice were performed with AB surgery to induce cardiac hypertrophy. Western blotting results showed that ZNF418 expression was significantly inhibited in hearts of ZNF418 KO mice compared with WT mice (Fig 3A). After AB treatment for 4 weeks, the ratios of HW/BW, LW/BW, HW/TL in either ZNF418 KO mice or WT mice were significantly higher than sham treatment, as well as were increased in AB-treated ZNF418 KO mice in comparison with AB-treated WT mice (all *P* < 0.05, Fig 3B). Echocardiography and hemodynamic measurements showed that AB treatment induced higher LVEDD and LVESD, but lower EF% and FS% than sham treatment (*P* < 0.05, Fig 3C). In addition, AB-treated ZNF418 KO mice exhibited remarkably increased LVEDD and LVESD, as well as decreased EF% and FS% in AB-treated WT mice (*P* < 0.05, Fig 3C). HE, WGA and PSR staining in cross sectional area revealed a significantly increased left ventricular cross-sectional area and a more significant fibrosis in the myocardium of AB-treated ZNF418 KO mice compared with AB-treated WT mice (Fig 3D and 3E). Consistently, the expression levels of fibrotic markers, including collagen 1a, collagen III, MMP-2 and Fibronection, were all obviously increased in AB-treated ZNF418 KO mice compared with AB-treated WT mice (all *P* < 0.05, Fig 3F). Furthermore, several hypertrophic markers were detected to confirm the effect of ZNF418 deficiency on cardiac hypertrophy. The results showed that the mRNA levels of ANP, BNP, β-MHC and MCIP1.4 were dramatically higher in ZNF418 KO mice than those in WT mice after AB treatment for 4 weeks (all *P* < 0.05, Fig 3G).

### Conditional overexpression of ZNF418 exhibits attenuated hypertrophic response *in vivo*

**Fig 4:**
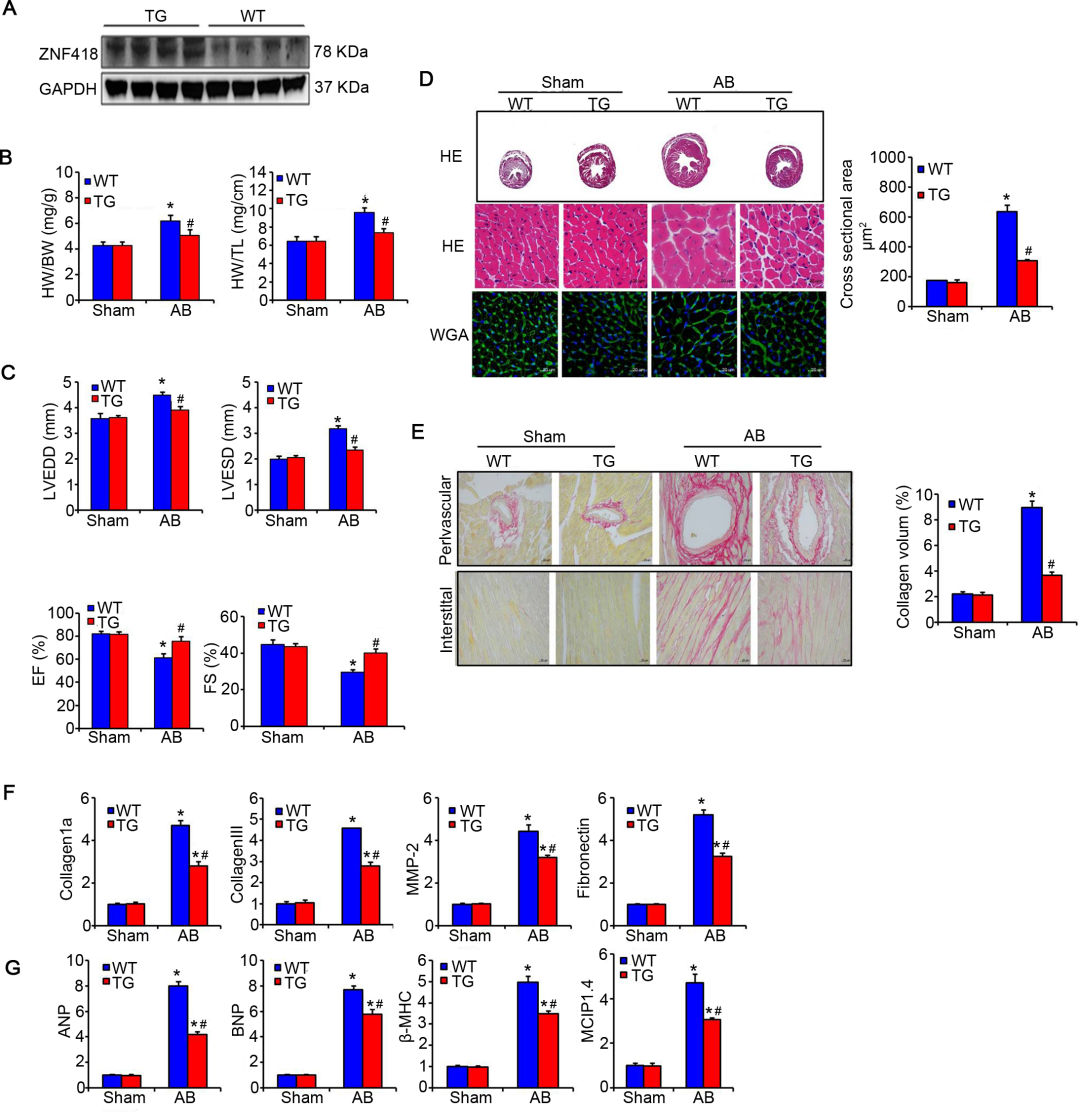
Overexpression of ZNF418 protects against aortic banding (AB)-induced hypertrophy. **(A)** The expression of ZNF418 in the hearts form wild-type (WT) and ZNF418 transgenic (TG) mice by western blotting; **(B)** Statistical results for the percentage of heart weight/body weight (HW/BW) and HW/tibial length (TL) (n = 13–15 for each group, *P < 0.05 vs. WT/sham; ^#^*P* < 0.05 vs. WT/AB); **(C)** Statistical results for the parameters of the echocardiographic results, including left ventricle end-diastolic dimension (LVEDD), left ventricle end-systolic diameter (LVESD), ejection fraction (EF) and fraction shortening (FS) in WT and TG mice after sham or AB surgery for 4 weeks (n = 6 mice per experimental group); **(D)** Histological analyses of the cross sectional area of hearts form WT and ZNF418 TG mice after sham or AB surgery for 4 weeks were stained with HE and WGA (n = 9 mice per group; scale bar, 100 μm for top HE staining, scale bar, 20 μm for middle HE staining and lower WGA staining); **(E)** Picrosirius red staining on histological sections from the left ventricles of WT and TG mice hearts after sham or AB surgery for 4 weeks (n = 7–8 mice per experimental group; scale bars, 20 μm.). The fibrotic areas of individual sections were quantified using Image-Pro Plus 6.0 software; **(F)** The mRNA expression levels of fibrotic markers-collagen 1a, collagen III, matrix metalloproteinase-2 (MMP-2), Fibronectin in the hearts of WT and TG mice were detected with real-time quantitative PCR after sham and AB surgery for 4 weeks (n = 5–7, *P < 0.05 vs. WT/sham or TG/sham, ^#^*P* < 0.05 vs. WT/AB); **(G)** The results of real-time quantitative PCR showed the mRNA levels of hypertrophy markers, including atrial natriuretic peptide (ANP), brain natriuretic peptide (BNP), β-myosin heavy chain (β-MHC) and modulatory calcineurin-interacting protein1.4 (MCIP1.4), in ZNF418 TG and WT mice hearts after AB or sham treatment for 4 weeks (n = 5 independent experiments, *P < 0.05 vs. WT/sham or TG/sham, ^#^*P* < 0.05 vs. WT/AB).

To further assess whether up-regulated ZNF418 could affect the hypertrophic response *in vivo*, ZNF418 TG mice were used for the following experiments. Four independent lines of ZNF418 TG mice was verified by western blotting and the results showed that ZNF418 expression was significantly increased in hearts of ZNF418 TG mice compared with WT mice (Fig 4A). ZNF418 TG or WT mice were treated with sham or AB surgery for 4 weeks. AB-treated ZNF418 TG mice showed lower ratios of HW/BW and HW/TL in contrast with WT mice (*P* < 0.05), indicating significantly alleviated hypertrophic response (Fig 4B). The echocardiography also showed a significant decrease in LVEDD and LVESD, as well as a markedly improvement in EF% and FS% in AB-treated ZNF418 TG mice compared with WT mice (all *P* < 0.05, Fig 4C). Meanwhile, HE and WGA analysis clearly showed that the myocyte size was obviously reduced in AB-treated ZNF418 TG mice compared to WT mice (*P* < 0.05, Fig 4D). In addition, PSR staining revealed that AB-treated ZNF418 TG mice had remarkably decreased collagen volume within the interstitial and perivascular spaces in comparison with WT mice (*P* < 0.05, Fig 4E), which were consistent with the results of reduced mRNA levels of collagen 1a, collagen III, MMP-2 and Fibronectin (all *P* < 0.05, Fig 4F). These data suggested that the fibrosis of hearts was decreased in ZNF418 TG mice after AB treatment for 4 weeks compared with WT mice. Furthermore, AB treatment significantly up-regulated the mRNA levels of ANP, BNP, β-MHC and MCIP1.4 in WT mice, while these expression was obviously inhibited in ZNF418 TG mice (all *P* < 0.05, Fig 4G).

### Reduced hypertrophic response induced by ZNF418 requires the activity of c-Jun/AP-1

**Fig 5:**
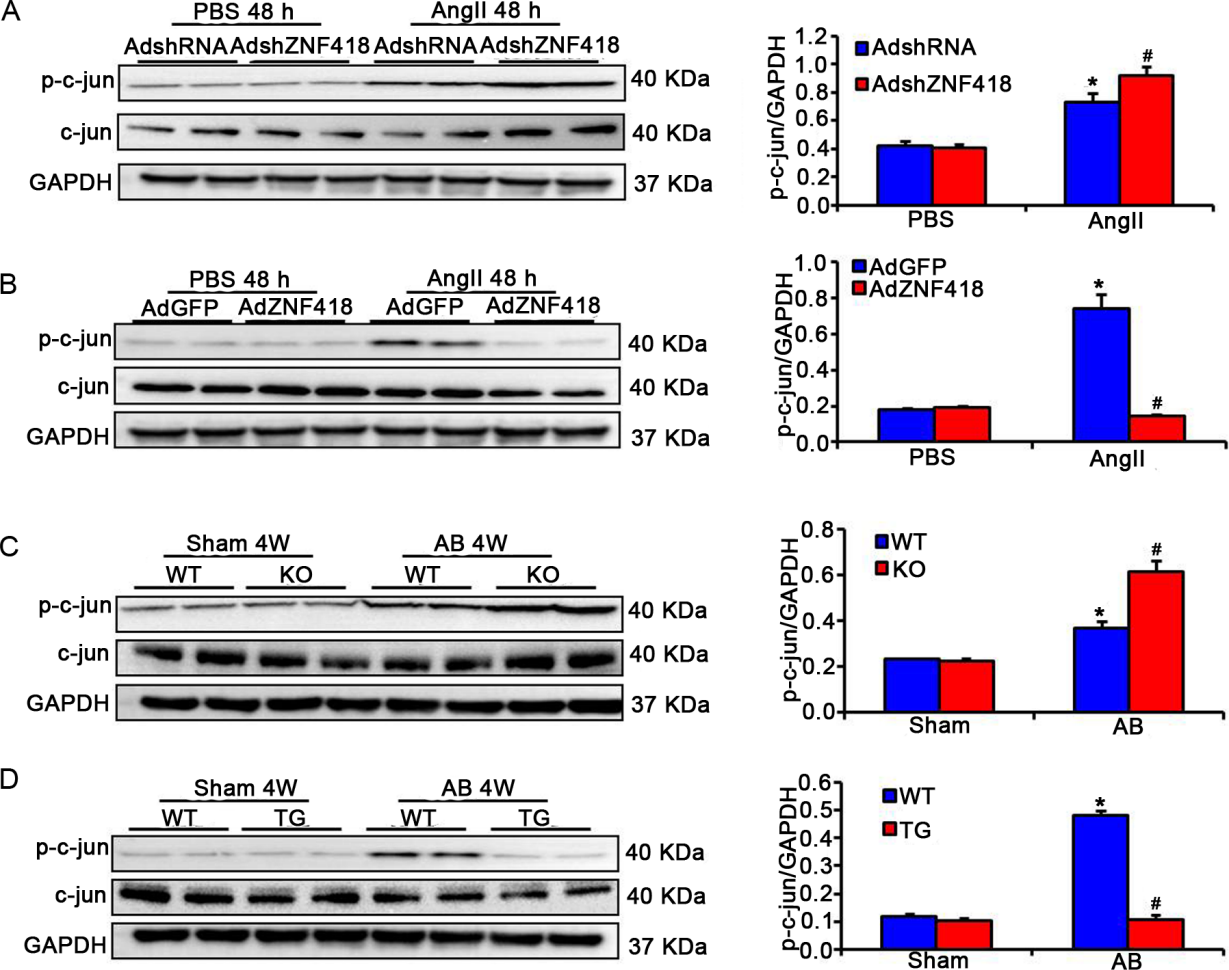
ZNF418 regulates c-Jun/AP-1 pathway in the heart. **(A)** The expressions of phosphorylated and total c-jun in samples of primary cardiomyocytes that were infected with AdshZNF418 or AdshRNA and then stimulated with angiotensin II (Ang II) for 48 h (n = 3, *P < 0.05 vs. AdshRNA; ^#^*P* < 0.05 vs. AdshRNA/Ang II); **(B)** The expressions of phosphorylated and total c-jun in samples of primary cardiomyocytes that were infected with AdZNF418 or AdGFP and then stimulated with Ang II for 48 h (n = 3, *P < 0.05 vs. AdGFP/PBS; ^#^*P* < 0.05 vs. AdGFP/Ang II); **(C)** The proteins levels of phosphorylated and total c-jun in samples from wild-type(WT) and ZNF418 KO mice after sham and AB treatment for 4 weeks (n = 3, *P < 0.05 vs. WT/sham; ^#^*P* < 0.05 vs. WT/AB); **(D)** The proteins levels of phosphorylated and total c-jun in samples from WT and ZNF418 transgenic (TG) mice after sham and AB treatment for 4 weeks (n = 3, *P < 0.05 vs. WT/sham; ^#^*P* < 0.05 vs. WT/AB).

To investigate the mechanism of reduced hypertrophic response induced by ZNF418, c-jun, one of protein constituents of AP-1 was examined by western blotting. The results suggested that the AngII-treated cardiomyocytes with AdshZNF418 showed dramatically enhanced the phosphorylation level of c-jun compared to cardiomyocytes with AdshRNA (*P* < 0.05, Fig 5A). On the contrary, AngII-induced the phosphorylation levels of c-jun was almost completely blocked in cardiomyocytes with AdZNF418 compared with cells with AdGFP (*P* < 0.05, Fig 5B). To further confirm the effect of ZNF418 on c-Jun/AP-1 pathway *in vivo*, the phosphorylation level of c-jun was detected in AB-treated ZNF418 KO, TG and WT mice. Consistent with the *in vitro* results, greater level of phosphorylated c-jun was observed in ZNF418 KO mice than that in WT mice (*P* < 0.05, Fig 5C). In contrast, the phosphorylation levels of c-jun was significantly attenuated in ZNF418 TG mice compared with WT mice (*P* < 0.05, Fig 5D).

## Discussion

In this study, our findings evidenced that ZNF418 was essential for resisting hypertrophic response of hearts. The expression of ZNF418 was markedly down-regulated in hearts of DCM or HCM patients, AB-induced cardiac hypertrophy mice, and Ang II- or PE-induced hypertrophic primary cardiomyocytes. Subsequently, we further demonstrated that down-regulated ZNF418 exacerbated the myocyte hypertrophy, perivascular and interstitial fibrosis, and (as well as) increased the mRNA levels of collagen 1a, collagen III, MMP-2, Fibronection, ANP, BNP, β-MHC and MCIP1.4 in hearts of AB-treated ZNF418 KO mice or Ang II-treated cardiomyocytes with AdshZNF418; conversely, hypertrophic responses were reduced in the ZNF418 TG mice treated by AB and the AdZNF418-transfected primary cardiomyocytes treated by Ang II. Additionally, the deficiency of ZNF418 enhanced the phosphorylation level of c-jun, and the ZNF418 overexpression suppressed the phosphorylation level of c-jun *in vivo* and *in vitro*.

KRAB/C2H2 zinc finger proteins had been demonstrated to involve in the regulation of many physiological or pathological processes, such as cell proliferation, apoptosis, and differentiation, embryonic development and oncogenesis [21]. Previous studies had demonstrated that both ZNF 382 and ZNF 480 might be play an important role in regulating cardiac development and pathogenesis [22,23]. The recently published paper also demonstrated that as a C2H2 protein, ZNF307 negatively regulate pressure overload-induced cardiac hypertrophy through inhibiting the NF-kB signaling [24]. Similarly, higher level of ZNF418 was found in heart compared with other tissues [15]. However, few studies had investigate the role of ZNF418 in cardiac hypertrophy. Interestingly, our study found that ZNF418 level was down-regulated in hearts of human or mice with cardiac hypertrophy as well as hypertrophic primary cardiomyocytes, which suggested that ZNF418 might be associated with cardiac hypertrophy. It had been reported that pressure overload such as AB could induce cardiac hypertrophy and fibrosis [25,26]. The high levels of ANP, BNP, β-MHC and MCIP1.4 had been demonstrated in cardiac hypertrophy or heart failure, and these genes were well-known as the hypertrophy markers in many studies [27–30]. Our study found that overexpression of ZNF418 significantly reduced ratios of HW/BW, LW/BW, HW/TL, the left ventricle-function, cardiomyocyte cross-sectional area and collagen accumulation in AB-treated ZNF418 TG mice compared with AB-operated WT mice. Meanwhile, the mRNA levels of the hypertrophy markers, including ANP, BNP, β-MHC and MCIP1.4, as well as fibrotic markers, containing collagen 1a and collagen III, MMP2 and Fibronectin, were reduced via the cardiac-specific overexpression of ZNF418, indicating that up-regulated ZNF418 might protect against cardiac hypertrophy and fibrosis caused by pressure overload. Conversely, we found that greatly increase of these hypertrophy responses and fibrosis was observed in ZNF418 KO mice. Furthermore, the effects of ectopic ZNF418 on Ang II-induced cardiomyocyte hypertrophy *in vitro* were consistent with the animal experiment results. All these results confirmed that ZNF418 was an important regulator in protecting the heart from pressure overload or AngII stimulation.

To elucidate the molecular mechanisms of ZNF418-mediated anti-hypertrophic effect, we focused on the changes of c-jun/AP-1 pathway. AP-1 was an important transcription factor regulated by various signaling pathways such as MAPK pathway [31]. MAPK pathway was considered as a vital intracellular signaling pathway in development of cardiac hypertrophy by regulating kinase activation, including extracellular-signal-regulated kinases (ERKs), p38 and c-jun N-terminal kinases (JNK) [32–34]. Previous study had shown that increased activities of ERK, p38 and JNK could exacerbate the development of cardiomyocyte hypertrophy [35,36]. Overexpression of AP-1 was reported to contribute the occurrence of heart failure [11]. In addition, increasing evidences had demonstrated that AP-1 activity was reduced in isolated myocardial cells and in heart tissue by neuro hormonal stimuli and pressure overload [37–39]. C-jun, as a major member of the AP-1, had been also previously exhibited to be upregulated in mechanical and pharmacological hypertrophic stimuli [40–42]. Notably, up-regulation of ZNF418 had been proved to reduce the activation of AP-1 [15]. Our study revealed that the overexpression of ZNF418 could suppress the phosphorylation level of c-jun *in vivo* and *in vitro*, while ZNF418 knockout dramatically enhanced the phosphorylation levels of c-jun. These data indicated that the anti-hypertrophic effect of ZNF418 might be associated with the activation of c-Jun/AP-1.

## Conclusion

The present study confirms the down-regulation of ZNF418 in cardiac hypertrophy and fibrosis. Overexpression of ZNF418 in adult hearts exhibits attenuated hypertrophic response including myocyte hypertrophy and fibrosis. Furthermore, ZNF418 may protect against the development of cardiac hypertrophy via inhibiting the activity of c-jun/AP-1.

## Supporting information

S1 Fig Generation of ZNF418-KO mice using the CRISPR/Cas9 system. **(A)** One sgRNA was designed and constructed to target exon 2 of the ZNF418 gene in mice. **(B)** Representative results of DNA sequencing from the founders, and the double peak traces in the sequencing chromatogram indicated an indel in heterozygous mutants. **(C)** The PCR products of the 4 mutant founders were TA cloned and sequenced so that the precise mutations of the indels could be verified. **(D)** Genotyping of ZNF418-KO (KO) mice via PCR and 3.0% agarose gel electrophoresis. A 137-bp band indicated the WT allele, and a 129-bp band indicated the mutated *ZNF418* allele.
(TIF)

S1 File The original uncropped and unadjusted blots. (RAR)
