# Supplementary figures and images for: Zinc-finger protein 418 overexpression protects against cardiac hypertrophy and fibrosis
Source: PLoS One. 2017 Oct 24;12(10):e0186635. doi: 10.1371/journal.pone.0186635 (PMC5655480; doi:10.1371/journal.pone.0186635)

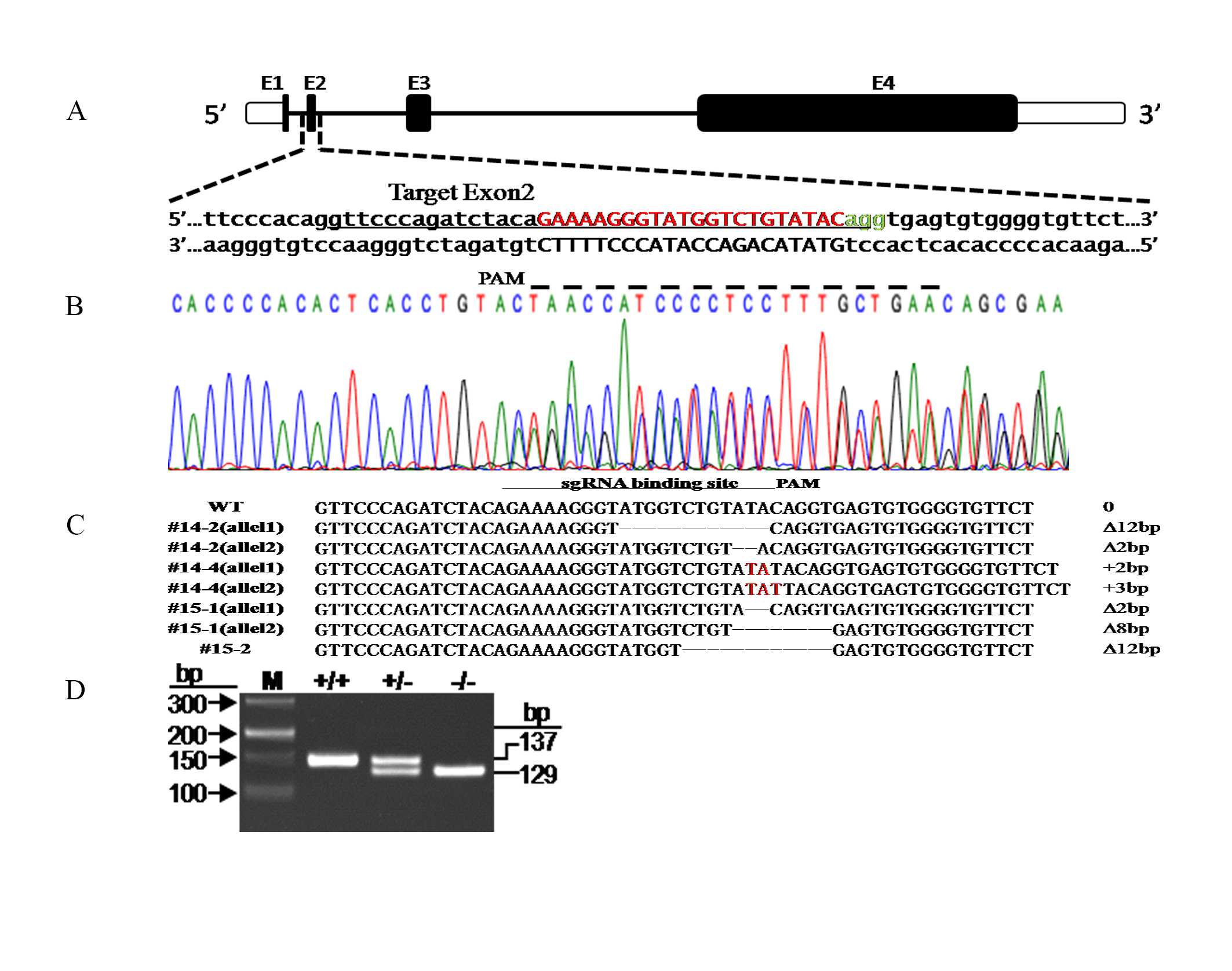

Supplement: S1 Fig — (A) One sgRNA was designed and constructed to target exon 2 of the ZNF418 gene in mice. (B) Representative results of DNA sequencing from the founders, and the double peak traces in the sequencing chromatogram indicated an indel in heterozygous mutants. (C) The PCR products of the 4 mutant founders were TA cloned and sequenced so that the precise mutations of the indels could be verified. (D) Genotyping of ZNF418-KO (KO) mice via PCR and 3.0% agarose gel electrophoresis. A 137-bp band indicated the WT allele, and a 129-bp band indicated the mutated ZNF418 allele. (TIF) [file pone.0186635.s001.tif]
